# Supplementary material for: A taxonomic outline of the Poecilimon affinis complex (Orthoptera) using the geometric morphometric approach
Source: PeerJ. 2021 Dec 22;9:e12668. doi: 10.7717/peerj.12668 (PMC8710050; doi:10.7717/peerj.12668)
Supplement: Supplemental Information 1 — Mahalanobis distances (bold) and Procrustes distances (narrow). [file peerj-09-12668-s001.docx]

Table S1:

Difference in tegmen shapes among species from the *P. ornatus* group with canonical variate analysis (CVA). Mahalanobis distances (bold) and

Procrustes distances (narrow).

| Species | *affinis* | *hoelzeli* | *jablanicensis* | *nobilis* | *nonveilleri* | *obesus* | *poecilus* | *p pseudornatus* |
| --- | --- | --- | --- | --- | --- | --- | --- | --- |
| *affinis* | **-** | 0.1243 | 0.0964 | 0.1790 | 0.0467 | 0.1347 | 0.0392 | 0.0323 |
| *hoelzeli* | **8.0504** | **-** | 0.1663 | 0.2703 | 0.1108 | 0.0705 | 0.1298 | 0.1341 |
| *jablanicensis* | **8.2179** | **14.1805** | **-** | 0.1857 | 0.0977 | 0.1774 | 0.1217 | 0.1091 |
| *nobilis* | **14.5668** | **18.8282** | **14.0134** | **-** | 0.1894 | 0.2788 | 0.1818 | 0.1745 |
| *nonveilleri* | **2.9027** | **8.4726** | **7.6440** | **14.6725** | **-** | 0.1267 | 0.0615 | 0.0515 |
| *obesus* | **9.2912** | **5.0108** | **14.9755** | **19.6637** | **9.3176** | **-** | 0.1369 | 0.1416 |
| *poecilus* | **3.2060** | **7.9251** | **9.5758** | **15.9968** | **4.7456** | **9.3763** | **-** | 0.0309 |
| *pseudornatus* | **2.5030** | **8.5373** | **8.7091** | **14.5359** | **3.6143** | **9.5615** | **2.7984** | - |
