# Supplementary material for: A taxonomic outline of the Poecilimon affinis complex (Orthoptera) using the geometric morphometric approach
Source: PeerJ. 2021 Dec 22;9:e12668. doi: 10.7717/peerj.12668 (PMC8710050; doi:10.7717/peerj.12668)
Supplement: Supplemental Information 2 — Mahalanobis distances (bold) and Procrustes distances (narrow). [file peerj-09-12668-s002.docx]

Table S2:

Difference in tegmen shapes among taxa from the *P. affinis* complex with canonical variate analysis (CVA). Mahalanobis distances (bold) and

Procrustes distances (narrow).

| Species | *a.affinis* | *a.dinaricus* | *a.hajlensis* | *a.komareki* | *rumijae* | *a.serbicus* | *nonveilleri* | *poecilus* | *pseudornatus* |
| --- | --- | --- | --- | --- | --- | --- | --- | --- | --- |
| *a.affinis* | **-** | 0.0848 | 0.0453 | 0.0884 | 0.0711 | 0.0537 | 0.0609 | 0.0547 | 0.0529 |
| *a.dinaricus* | **5.9991** | **-** | 0.0732 | 0.1100 | 0.1161 | 0.0880 | 0.0968 | 0.0878 | 0.0816 |
| *a.hajlensis* | **4.0575** | **5.7681** | **-** | 0.0739 | 0.0747 | 0.0513 | 0.0682 | 0.0485 | 0.0477 |
| *a.komareki* | **6.2092** | **8.1340** | **5.6483** | **-** | 0.0741 | 0.0629 | 0.0843 | 0.0750 | 0.0691 |
| *rumijae* | **3.9873** | **7.0199** | **4.9784** | **5.3684** | **-** | 0.0537 | 0.0601 | 0.0657 | 0.0575 |
| *a.serbicus* | **3.8398** | **6.8326** | **4.6652** | **6.1018** | **4.6009** | **-** | 0.0448 | 0.0361 | 0.0251 |
| *nonveilleri* | **3.6498** | **7.1259** | **4.1816** | **5.6240** | **4.3468** | **3.9114** | **-** | 0.0615 | 0.0515 |
| *poecilus* | **3.8299** | **6.3236** | **4.5367** | **6.9142** | **4.7404** | **3.6537** | **4.7649** | **-** | 0.0309 |
| *pseudornatus* | **3.3300** | **6.72220** | **4.4654** | **6.4675** | **4.2757** | **3.0901** | **3.7281** | **2.7717** | - |
