# Supplementary material for: A taxonomic outline of the Poecilimon affinis complex (Orthoptera) using the geometric morphometric approach
Source: PeerJ. 2021 Dec 22;9:e12668. doi: 10.7717/peerj.12668 (PMC8710050; doi:10.7717/peerj.12668)
Supplement: Supplemental Information 3 — Mahalanobis distances (bold) and Procrustes distances (narrow). [file peerj-09-12668-s003.docx]

Table S3:

Difference in ovipositor shapes among species from the *P. ornatus* group with canonical variate analysis (CVA). Mahalanobis distances (bold) and Procrustes distances (narrow).

| Species | *affinis* | *ampliatus* | *artedentatus* | *gracilis* | *hoelzeli* | *jablanicensis* | *nobilis* | *nonveilleri* | *obesus* | *poecilus* | *pseudornatus* |
| --- | --- | --- | --- | --- | --- | --- | --- | --- | --- | --- | --- |
| *affinis* | **-** | 0.0949 | 0.1139 | 0.1473 | 0.0351 | 0.0969 | 0.1437 | 0.0720 | 0.1004 | 0.0472 | 0.0637 |
| *ampliatus* | **6.0532** | **-** | 0.0954 | 0.0956 | 0.0713 | 0.0797 | 0.1117 | 0.1072 | 0.1078 | 0.0881 | 0.1336 |
| *artedentatus* | **8.4370** | **7.7347** | **-** | 0.1169 | 0.1026 | 0.0917 | 0.0718 | 0.0795 | 0.0397 | 0.0748 | 0.1299 |
| *gracilis* | **9.0042** | **8.9659** | **12.7381** | **-** | 0.1235 | 0.1159 | 0.1108 | 0.1565 | 0.1387 | 0.1345 | 0.1941 |
| *hoelzeli* | **3.0805** | **5.4878** | **7.0018** | **9.5072** | **-** | 0.0789 | 0.1273 | 0.0711 | 0.0940 | 0.0467 | 0.0829 |
| *jablanicensis* | **9.5259** | **10.4518** | **7.7488** | **14.1705** | **8.9512** | **-** | 0.0932 | 0.0920 | 0.0899 | 0.0802 | 0.1315 |
| *nobilis* | **11.9581** | **10.5757** | **5.4908** | **15.7156** | **10.7249** | **7.4292** | **-** | 0.1230 | 0.0859 | 0.1110 | 0.1679 |
| *nonveilleri* | **5.7586** | **6.5558** | **6.1878** | **11.7449** | **4.2352** | **8.1217** | **9.6513** | **-** | 0.0495 | 0.0381 | 0.0704 |
| *obesus* | **6.9948** | **6.7869** | **3.0230** | **12.0451** | **5.5767** | **7.1694** | **6.6624** | **3.6954** | **-** | 0.0555 | 0.1068 |
| *poecilus* | **3.5589** | **5.5663** | **5.7738** | **10.0441** | **2.7815** | **8.6736** | **9.6064** | **3.9132** | **4.0711** | **-** | 0.0722 |
| *pseudornatus* | **4.4070** | **7.7901** | **8.0642** | **12.0775** | **4.5740** | **10.4786** | **11.9396** | **5.7617** | **6.6893** | **4.1426** | - |
