# Supplementary material for: A taxonomic outline of the Poecilimon affinis complex (Orthoptera) using the geometric morphometric approach
Source: PeerJ. 2021 Dec 22;9:e12668. doi: 10.7717/peerj.12668 (PMC8710050; doi:10.7717/peerj.12668)
Supplement: Supplemental Information 4 — Mahalanobis distances (bold) and Procrustes distances (narrow). [file peerj-09-12668-s004.docx]

Table S4:

Difference in ovipositor shapes among taxa from the *P. affinis* complex with canonical variate analysis (CVA). Mahalanobis distances (bold) and Procrustes distances (narrow).

| Species | *a.affinis* | *a.dinaricus* | *a.hajlensis* | *a.komareki* | *rumijae* | *a.serbicus* | *nonveilleri* | *poecilus* | *pseudornatus* |
| --- | --- | --- | --- | --- | --- | --- | --- | --- | --- |
| *a.affinis* | **-** | 0.0656 | 0.0685 | 0.1349 | 0.0356 | 0.0319 | 0.0824 | 0.0603 | 0.0714 |
| *a.dinaricus* | **6.8512** | **-** | 0.1008 | 0.1542 | 0.0924 | 0.0745 | 0.1005 | 0.0928 | 0.0882 |
| *a.hajlensis* | **8.9269** | **14.4906** | **-** | 0.1523 | 0.0881 | 0.0460 | 0.0847 | 0.0596 | 0.1099 |
| *a.komareki* | **6.3547** | **5.2708** | **13.9868** | **-** | 0.1346 | 0.1441 | 0.0847 | 0.1311 | 0.1027 |
| *rumijae* | **2.6873** | **7.4340** | **10.2852** | **5.7461** | **-** | 0.0531 | 0.0860 | 0.0652 | 0.0678 |
| *a.serbicus* | **3.2550** | **8.4123** | **7.1306** | **8.2658** | **4.9003** | **-** | 0.0679 | 0.0419 | 0.0844 |
| *nonveilleri* | **7.6163** | **8.9126** | **10.8068** | **9.1799** | **9.0290** | **6.2751** | **-** | 0.0392 | 0.0705 |
| *poecilus* | **5.2135** | **7.8453** | **8.8232** | **7.2700** | **6.2663** | **4.1432** | **4.1693** | **-** | 0.0755 |
| *pseudornatus* | **7.2647** | **5.3820** | **14.5025** | **4.4960** | **7.1256** | **9.6129** | **10.1917** | **6.4303** | - |
