# Supplementary material for: A taxonomic outline of the Poecilimon affinis complex (Orthoptera) using the geometric morphometric approach
Source: PeerJ. 2021 Dec 22;9:e12668. doi: 10.7717/peerj.12668 (PMC8710050; doi:10.7717/peerj.12668)
Supplement: Supplemental Information 5 — Mahalanobis distances (bold) and Procrustes distances (narrow). [file peerj-09-12668-s005.docx]

Table S5:

Difference in male cercus shapes among species from the *P. ornatus* group with canonical variate analysis (CVA). Mahalanobis distances (bold) and Procrustes distances (narrow).

| Species | *affinis* | *hoelzeli* | *jablanicensis* | *nobilis* | *nonveilleri* | *obesus* | *poecilus* | *pseudornatus* |
| --- | --- | --- | --- | --- | --- | --- | --- | --- |
| *affinis* | **-** | 0.1341 | 0.0886 | 0.1529 | 0.0563 | 0.0632 | 0.0350 | 0.0339 |
| *hoelzeli* | **8.3755** | **-** | 0.1349 | 0.0814 | 0.0928 | 0.1437 | 0.1373 | 0.1426 |
| *jablanicensis* | **8.7027** | **12.2488** | **-** | 0.1519 | 0.1064 | 0.1186 | 0.1001 | 0.0817 |
| *nobilis* | **10.5209** | **9.0083** | **11.2681** | **-** | 0.1129 | 0.1459 | 0.1565 | 0.1706 |
| *nonveilleri* | **4.1064** | **7.2670** | **10.7513** | **10.7568** | **-** | 0.0792 | 0.0671 | 0.0757 |
| *obesus* | **6.5412** | **10.3968** | **9.3670** | **8.1348** | **8.5007** | **-** | 0.0443 | 0.0898 |
| *poecilus* | **3.1067** | **9.1822** | **7.8319** | **10.4994** | **5.1264** | **6.0240** | **-** | 0.0587 |
| *pseudornatus* | **2.7073** | **8.8268** | **8.9629** | **11.6876** | **5.3179** | **8.0193** | **4.1552** | - |
