# Supplementary material for: A taxonomic outline of the Poecilimon affinis complex (Orthoptera) using the geometric morphometric approach
Source: PeerJ. 2021 Dec 22;9:e12668. doi: 10.7717/peerj.12668 (PMC8710050; doi:10.7717/peerj.12668)
Supplement: Supplemental Information 6 — Mahalanobis distances (bold) and Procrustes distances (narrow). [file peerj-09-12668-s006.docx]

Table S6:

Difference in male cercus shapes among taxa from the *P. affinis* complex with canonical variate analysis (CVA). Mahalanobis distances (bold) and Procrustes distances (narrow).

| Species | *a.affinis* | *a.dinaricus* | *a.hajlensis* | *a.komareki* | *rumijae* | *a.serbicus* | *nonveilleri* | *poecilus* | *pseudornatus* |
| --- | --- | --- | --- | --- | --- | --- | --- | --- | --- |
| *a.affinis* | **-** | 0.0634 | 0.0318 | 0.0534 | 0.0304 | 0.0376 | 0.0569 | 0.0301 | 0.0391 |
| *a.dinaricus* | **7.2368** | **-** | 0.0690 | 0.0977 | 0.0666 | 0.0682 | 0.0402 | 0.0711 | 0.0842 |
| *a.hajlensis* | **3.3261** | **8.3160** | **-** | 0.0696 | 0.0463 | 0.0323 | 0.0541 | 0.0481 | 0.0315 |
| *a.komareki* | **4.6509** | **8.6480** | **5.4297** | **-** | 0.0436 | 0.0749 | 0.0985 | 0.0555 | 0.0535 |
| *rumijae* | **3.7141** | **6.9099** | **4.9815** | **3.4088** | **-** | 0.0440 | 0.0680 | 0.0461 | 0.0416 |
| *a.serbicus* | **3.1956** | **8.1597** | **3.1122** | **5.5398** | **4.9102** | **-** | 0.0494 | 0.0508 | 0.0453 |
| *nonveilleri* | **4.3693** | **5.7994** | **5.4986** | **6.7563** | **5.5340** | **5.0108** | **-** | 0.0645 | 0.0756 |
| *poecilus* | **3.4275** | **8.6212** | **3.7312** | **5.2959** | **5.0327** | **3.1874** | **5.4729** | **-** | 0.0585 |
| *pseudornatus* | **3.4136** | **8.3486** | **2.8732** | **4.7826** | **4.4681** | **4.2193** | **5.8261** | **4.6717** | - |
