# Supplementary material for: A taxonomic outline of the Poecilimon affinis complex (Orthoptera) using the geometric morphometric approach
Source: PeerJ. 2021 Dec 22;9:e12668. doi: 10.7717/peerj.12668 (PMC8710050; doi:10.7717/peerj.12668)
Supplement: Supplemental Information 7 — Mahalanobis distances (bold) and Procrustes distances (narrow). [file peerj-09-12668-s007.docx]

Table S7:

Difference in pronotum shapes among species from the *P. ornatus* group with canonical variate analysis (CVA). Mahalanobis distances (bold) and Procrustes distances (narrow).

| Species | *affinis* | *gracilis* | *hoelzeli* | *jablanicensis* | *nobilis* | *nonveilleri* | *obesus* | *poecilus* | *pseudornatus* |
| --- | --- | --- | --- | --- | --- | --- | --- | --- | --- |
| *affinis* | **-** | 0.1120 | 0.0400 | 0.0692 | 0.1110 | 0.0818 | 0.1398 | 0.0286 | 0.0754 |
| *gracilis* | **8.7757** | **-** | 0.1338 | 0.1591 | 0.1545 | 0.1011 | 0.0286 | 0.1140 | 0.1332 |
| *hoelzeli* | **2.7954** | **10.3568** | **-** | 0.0697 | 0.1030 | 0.0972 | 0.1320 | 0.0533 | 0.0815 |
| *jablanicensis* | **4.1802** | **11.4995** | **4.9195** | **-** | 0.1098 | 0.1365 | 0.1495 | 0.0800 | 0.0880 |
| *nobilis* | **5.9322** | **12.2787** | **6.0601** | **5.5256** | **-** | 0.1587 | 0.1135 | 0.1167 | 0.0953 |
| *nonveilleri* | **3.5627** | **7.9683** | **4.1678** | **6.3727** | **7.6699** | **-** | 0.1544 | 0.0762 | 0.1090 |
| *obesus* | **7.5493** | **12.8096** | **7.3672** | **8.1186** | **5.2972** | **8.7641** | **-** | 0.1386 | 0.0875 |
| *poecilus* | **2.2038** | **9.7049** | **3.4030** | **4.1163** | **5.5951** | **4.2377** | **7.7369** | **-** | 0.0727 |
| *pseudornatus* | **3.7020** | **9.9918** | **4.5955** | **4.4239** | **5.0230** | **5.7472** | **6.5642** | **3.7766** | **-** |
