# Supplementary material for: A taxonomic outline of the Poecilimon affinis complex (Orthoptera) using the geometric morphometric approach
Source: PeerJ. 2021 Dec 22;9:e12668. doi: 10.7717/peerj.12668 (PMC8710050; doi:10.7717/peerj.12668)
Supplement: Supplemental Information 8 — Mahalanobis distances (bold) and Procrustes distances (narrow). [file peerj-09-12668-s008.docx]

Table S8:

Difference in pronotum shapes among taxa from the *P. affinis* complex with canonical variate analysis (CVA). Mahalanobis distances (bold) and Procrustes distances (narrow).

| Species | *a.affinis* | *a.dinaricus* | *a.hajlensis* | *a.komareki* | *rumijae* | *a.serbicus* | *nonveilleri* | *poecilus* | *pseudornatus* |
| --- | --- | --- | --- | --- | --- | --- | --- | --- | --- |
| *a.affinis* | **-** | 0,0884 | 0,0474 | 0,0445 | 0,0622 | 0,0784 | 0,0800 | 0,0369 | 0,0837 |
| *a.dinaricus* | **4,3943** | **-** | 0,0650 | 0,0954 | 0,1044 | 0,0886 | 0,1128 | 0,0825 | 0,0964 |
| *a.hajlensis* | **2,7308** | **4,0142** | **-** | 0,0487 | 0,0891 | 0,0609 | 0,0776 | 0,0470 | 0,0569 |
| *a.komareki* | **4,1060** | **5,2404** | **3,4003** | **-** | 0,0754 | 0,0899 | 0,0893 | 0,0518 | 0,0757 |
| *rumijae* | **3,3874** | **4,0739** | **4,0658** | **5,1072** | **-** | 0,1261 | 0,1351 | 0,0743 | 0,1122 |
| *a.serbicus* | **3,1575** | **4,9320** | **3,3360** | **4,7335** | **4,8939** | **-** | 0,0695 | 0,0613 | 0,0836 |
| *nonveilleri* | **3,5199** | **5,0842** | **3,7839** | **4,7645** | **5,6766** | **4,0796** | **-** | 0,0762 | 0,1091 |
| *poecilus* | **3,1391** | **3,9590** | **3,6948** | **4,3128** | **3,2625** | **2,9688** | **4,6044** | **-** | 0,0727 |
| *pseudornatus* | **4,6006** | **5,3384** | **3,4472** | **4,4488** | **4,5959** | **4,1093** | **5,9363** | **3,8378** | - |
